# Roll-to-Roll Gravure Coating of PVDF on a Battery Separator for the Enhancement of Thermal Stability

**DOI:** 10.3390/polym15204108

**Published:** 2023-10-16

**Authors:** Gyuyoung Kim, Jin-Hee Noh, Horim Lee, Jaehak Shin, Dongjin Lee

**Affiliations:** 1 Department of Mechanical Design and Production Engineering, Konkuk University, Seoul 05029, Republic of Korea; 2 Daegu Technopark, Daegu 41256, Republic of Korea; 3 School of Mechanical and Aerospace Engineering, Konkuk University, Seoul 05029, Republic of Korea

**Keywords:** roll-to-roll (R2R), gravure coating, polyvinylidene fluoride (PVDF), battery separator, nonsolvent-induced phase separation (NIPS)

## Abstract

The polyethylene lithium-ion battery separator is coated with a polymer by means of a roll-to-roll (R2R) gravure coating scheme to enhance the thermal stability. The polyvinylidene fluoride (PVDF) or polyvinylidene fluoride-co-hexafluoropropylene (PVDF-HFP) is gravure-coated, and the pores are fabricated based on online nonsolvent-induced phase separation (NIPS). N-methylpyrrolidone is used as a solvent, and deionized water or a methanol mixture thereof is exploited as a nonsolvent in NIPS. Scanning electron microscopy confirms that the polymer film is formed and that the pores are well developed. The thermal shrinkage decreased by 20.0% and 23.2% compared to that of the bare separator due to the coating of PVDF and PVDF-HFP, respectively. The R2R gravure coating scheme is proven to be fully functional to tailor the properties of lithium-ion battery separators.

## 1. Introduction

Lithium-ion batteries are currently widely used in portable electronics, electric vehicles, and energy storage systems. The main components of a typical lithium-ion battery include the cathode, anode, electrolyte, and separator [1]. Of those, the separator plays an important role as a physical barrier between the anode and cathode [2]. It prevents direct contact, thereby inhibiting short circuits and thermal runaway, which can lead to catastrophic failures. As a permeable membrane, on the other hand, the separator allows the passage of lithium ions during the charge and discharge processes. The controlled lithium-ion transport via the separator ensures efficient and safe battery operation, maintains electrode integrity, and prevents dendrite formation.

Commercial polyolefin-based separators are polyethylene (PE), polypropylene (PP), and blends of PE and PP [3]. Even though they have relatively good mechanical properties and good chemical stability, they suffer from a relatively low melting point and potential thermal instability [4]. The low melting point of 115~135 °C, depending on the crystallinity in the case of PE, causes thermal shrinkage and eventually melts at elevated temperatures. This augments the risk of short circuits and thermal runaway in the lithium-ion battery. For practical applications, the separator should be dimensionally stable over a wide range of temperatures, and the thermal shrinkage should not exceed a certain level, e.g., 5% at 90 °C for 60 min [5].

The approaches to increasing the thermal stability of PE separators include compositing them with thermally resistant polymers [6,7,8,9,10] and ceramic materials [3,11,12]. The polymer coating is advantageous since it is simple; so, it can be incorporated into the production of the separator and is easy to mass-produce. The drawback of the polymer coating is that it allows adequate porosity without deteriorating the ionic passage in the PE separator itself. Consequently, an additional pore formation mechanism is necessary. In the case of ceramic coating, nanostructured entities such as nanoparticles, nanowires, nanopallets, etc., have been utilized to preserve the porosity of the separator. The ceramic materials that have been used include silica (SiO_2_), alumina (Al_2_O_3_), and zirconia (ZrO_2_). The drawback of this approach is that the nanostructured ceramic materials desquamate for some reason. Therefore, it is important for the nanostructured ceramic materials to be grafted firmly onto the separator. In terms of the coating method, most works [13,14,15] have been conducted with dip coating, where a small piece of separator was used. The separators were withdrawn from the coating solution manually or in a controlled manner, which cannot be mass-produced. Current research on a battery separator coating using either material focuses on enhancing the battery performance, safety, and lifespan via innovative coating materials and techniques.

The roll-to-roll continuous process has been widely used for the production of solar cells [16,17,18], organic light-emitting diodes [19,20], batteries [21], and transparent conductive electrodes [22,23,24] toward low-cost large-area electronics. It consists of several rollers, between which the various operations can be performed while the substrate, called the web, is transported continuously. In particular, solution-based operations of printing and coating have been demonstrated successfully for the production of electronics and functional layers. The web is controlled to have a certain speed and tension force that influence the cost and quality of the product. It is easy to mass-produce due to its high speed and large-area capacity. Furthermore, the manufacturing scheme can be readily altered by adding sub-units such as encapsulation [25], lamination [16], and calendaring [26].

In this work, the roll-to-roll (R2R) continuous mass production scheme was applied to coat a thermally resistant polymer on the PE separator. As an additional pore-formation mechanism, nonsolvent-induced phase separation (NIPS) was adopted for the R2R scheme. The materials were selected rationally based on the Hansen solubility parameters in order to produce functional polymer-coated PE separators. To demonstrate the versatility of R2R gravure coating, two kinds of polyvinylidene fluoride (PVDF) were used. The morphology and pore formation were investigated using scanning electron microscopy. In addition, the thermal shrinkage rate was evaluated based on area comparisons before and after the heat treatment. As a result, reduced thermal shrinkage was observed while preserving the porosity of the PE separators.

## 2. Materials and Methods

### 2.1. Materials

PVDF and polyvinylidene fluoride-co-hexafluoropropylene (PVDF-HFP) were R2R-coated in this work. PVDF powder (44080, Alfa Aesar, Ward Hill, MA, USA) and PVDF-HFP pellets (Kynar Flex^®^ 2800-00, Arkema, Colombes, France) were used as homopolymer and copolymer, respectively. The PVDF-HFP is a fluorinated thermoplastic copolymer with good mechanical and thermomechanical properties. N-methylpyrrolidone (NMP, 99.5%, Daejung Chemical Inc., Goryeong, Republic of Korea) was used as a solvent for PVDF and PVDF-HFP. Methanol (99.5%, Daejung Chemical Inc., Goryeong, Republic of Korea) and deionized water were used as coagulants in NIPS. A wet-processed polyethylene (PE) separator was obtained from Liaoyuan Hongtu Lithium-Ion Battery Separator Technology Co. (Zhuhai, China) and was used in the R2R coating process.

The coating solutions were prepared via magnetic stirring at room temperature for 24 h. It should be noted that the homopolymer PVDF is difficult to dissolve at a high concentration at room temperature and that the PVDF solution in NMP is highly viscous. However, copolymer PVDF-HFP had higher solubility in NMP than the homopolymer. Consequently, the PVDF solution was prepared to have 10.0 wt% concentration, while PVDF-HFP solutions were prepared to yield various concentrations of 10.0, 12.5, 15.0, and 17.5 wt% [27]. As nonsolvent coagulation liquid, the mixtures of methanol/DI water were prepared and stored at room temperature for at least 2 days prior to coating operation because of the heat generated when being mixed.

### 2.2. R2R Gravure Coating

**Figure 1:**
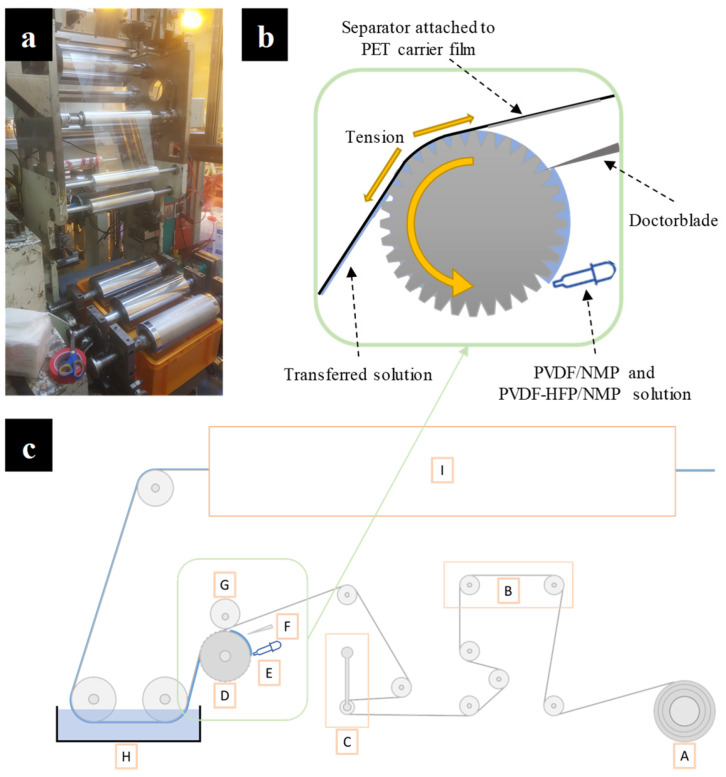
Roll-to-roll (R2R) gravure coating of PVDF on the battery separator: (**a**) digital image of the R2R gravure coating followed by the NIPS module and the schematics of (**b**) the gravure coating and (**c**) the whole R2R coating machine (A: unwinder, B: edge position controller, C: tension controller, D: gravure coating roll, E: solution feeder, F: doctoring blade, G: nip roll, H: coagulation bath, and I: dryer).

The gravure coating was carried out using an in-house R2R gravure printing/coating machine (Konkuk Univ.), as shown in Figure 1. The printing/coating machine depicted in Figure 1a functions by changing the roll depending on the operation. The diagonally inscribed micro-gravure roll (type M-100, line interval 254 μm, Media Engineering Inc., Chungju, Republic of Korea) in Figure 1b was soaked with the PVDF solution that was transferred onto the separator upon contact. Figure 1c shows the schematic of whole R2R gravure coating machine composed of an unwinder (A), an edge position controller (B), a tension controller (C), a gravure coating roll (D), a solution feeder (E), a doctoring blade (F), a nip roll (G), a coagulation bath (H), and a dryer (I). To exploit NIPS online, a coagulation bath of 20 L was added between the gravure coating roll and the dryer. A polyethylene terephthalate (PET) carrier film with a width of 300 mm was supplied from the unwinder. Throughout the R2R coating process, the speed of the carrier film was maintained at 1 m/min. Bare PE separators were tightly attached on the PET carrier film without wrinkles. PVDF and PVDF-HFP coating solutions were supplied onto the gravure coating roll, and the excess solution outside the patterned grooves was removed via the doctor blade. Unlike the printing operation [28], the nip roll was not involved, and the coating process was controlled by the tension force in the PET carrier film. It was maintained at 9 kgf to ensure the successful transfer of the coating solution onto the separators. After the separators were coated with the solution, they entered a coagulation bath filled with a nonsolvent to perform NIPS. The coagulation nonsolvent was deionized water (DI water) and a methanol/DI water mixture for the PVDF-HFP and PVDF solutions, respectively. Finally, the coated separators were dried in a convective oven at 60 °C. The coated separators were detached from the carrier film after drying and stored at ambient environment for characterization.

### 2.3. Characterization of the Coated Separator

The morphology of the bare and coated separators was characterized using field emission scanning electron microscopy (FE-SEM, SU8010, Hitachi, Tokyo, Japan). To evaluate the thermal stability of the coated separators, the size of the separators was compared before and after heat treatment. The heat treatment was conducted as follows. The coated separators were cut into 50 mm by 50 mm and scanned to obtain digital images. The convection oven was set to 130 °C, and the coated separators were placed in the oven for 30 min. After the coated separators were taken out, they were left in an ambient environment to cool down. The coated separators were digitally scanned again, and the size was compared to that before the thermal test. The digital images were converted to black and white images, and the number of pixels corresponding to the area of separators was counted using open-source image processing software (ImageJ 1.52). The shrinkage rate was calculated as the ratio of the number of pixels after heat treatment to the number of pixels before heat treatment [29].

## 3. Results and Discussion

### 3.1. Mechanism of Nonsolvent-Induced Phase Separation (NIPS)

An NIPS is one of the fastest techniques for producing a porous coating membrane [30]. It depends on a delicate interplay of solvents, polymers, and nonsolvents. In NIPS, the chosen solvent must have high solubility for the polymer, while the nonsolvent should possess the opposite property. Importantly, the solvent and nonsolvent should be readily miscible. In this study, PVDF was dissolved in NMP and applied in a liquid state onto a battery separator using the R2R coating process. When the coated separator submerges into a coagulation bath filled with nonsolvent, DI water or mixture of DI water/methanol, the solvent gradually separates from the PVDF film, allowing the nonsolvent to permeate the coated PVDF film. This liquid exchange induces a rapid decrease in the solubility of the PVDF, resulting in coagulation and the formation of pores within the coated film. This coagulation process typically occurs within a short period of time, rendering it highly compatible with the R2R manufacturing scheme.

The materials chosen in this study were based on the Hansen solubility parameters of *δ_d_*, *δ_p_*, and *δ_h_*, indicating the dispersion force, polar force, and hydrogen bonding force, respectively [31]. The interaction radius (*R*) is used for evaluating the solubility of the polymer in liquid:

(1) $$R^{2}={\left(2\Delta \delta_{d}\right)}^{2}+{\left(\Delta \delta_{p}\right)}^{2}+{\left(\Delta \delta_{h}\right)}^{2}.$$

The solubility of the polymer in liquid is inversely proportional to the R value. Table 1 demonstrates the Hansen solubility parameters for the PVDF, NMP, and nonsolvents (water and methanol) used in this study. The calculated R values, illustrating the compatibility between PVDF and liquids, are provided in Table 2. It is evident that PVDF exhibits good solubility in NMP, whereas its solubility is considerably reduced in both water and methanol. Thus, we chose NMP as a solvent, paired with either water or methanol as nonsolvents for the R2R gravure coating process in this work. Moreover, the characteristics of mixing between these liquids can be compared using the Hansen solubility parameters shown in Table 2. The NMP exhibits greater compatibility with methanol than with water due to its lower R value.

### 3.2. R2R Gravure-Coated Separator

**Figure 2:**
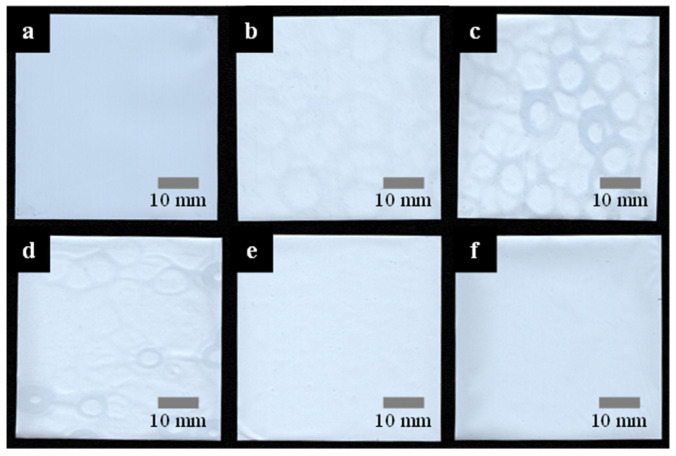
Digitally scanned images of battery separators: (**a**) bare and PVDF-coated separators as results of nonsolvent-induced phase separation (NIPS) in a methanol/DI water mixture of (**b**) 0, (**c**) 25, (**d**) 50, (**e**) 75, and (**f**) 100%.

The coating of the PVDF followed by NIPS was successfully performed on the basis of R2R. Figure 2 displays the digitally scanned images of battery separators coated with PVDF (10 wt%) followed by NIPS in a coagulation bath of various methanol concentrations along with a bare separator as a control. The coated separators appear marginally brighter than their bare counterpart, owing to the presence of the PVDF layer. While the bare separator in Figure 2a demonstrates a clean and smooth surface, the coated separators in Figure 2b–f exhibit rounded shapes, which is attributed to either the uneven application of PVDF during the coating process or the inherent variability in the surface characteristics stemming from the wetting and drying nature of the R2R coating process.

**Figure 3:**
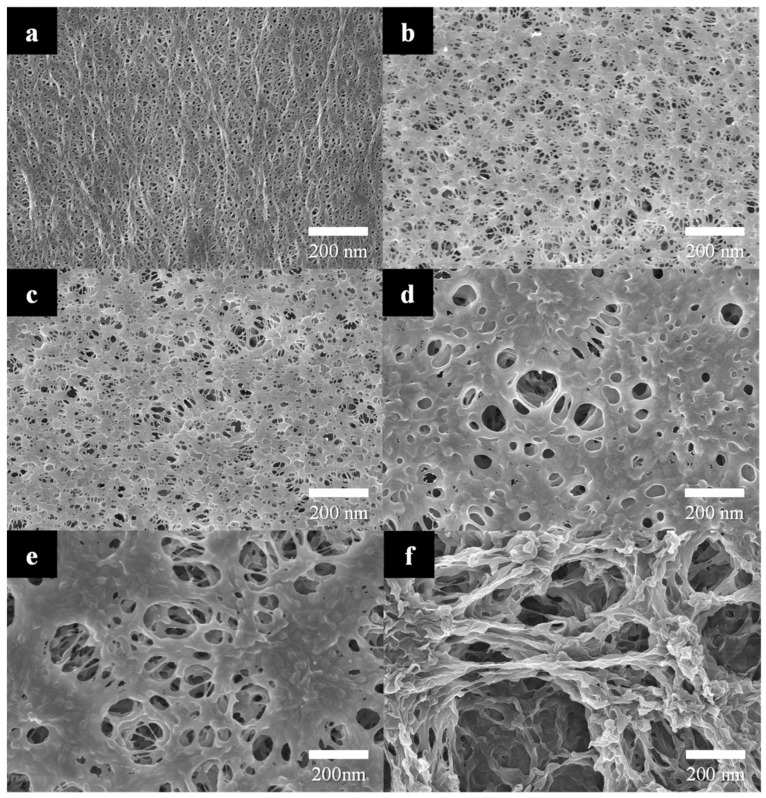
FE-SEM images of the separators: (**a**) bare and PVDF- (10 wt%) coated separators after NIPS in methanol/DI water of (**b**) 0, (**c**) 25, (**d**) 50, (**e**) 75, and (**f**) 100%.

FE-SEM images of the bare and PVDF-coated separators are shown in Figure 3. The bare separator predominantly exhibits fibrous structures. The pores among these fibers play a crucial role as the ionic pathways in lithium-ion battery. As the methanol concentration in the coagulation bath increases, the number of pores formed on the PVDF layer tends to decrease, and the size of the pores increases. At a methanol concentration of 0~25%, uniformly sized pores are evenly distributed across the surface, which positively affects the ionic conduction. With a further increase in the methanol concentration, however, the size of pores tends to increase as shown in Figure 3d,e. Ultimately the fibric structure of PVDF is observed in Figure 3f at a methanol concentration of 100%. This transformation can be attributed to the calculated interaction radius (R), which is larger for NMP–water than for NMP–methanol. Since methanol has higher miscibility with NMP than water, liquid exchange occurs at a higher rate, resulting in the formation of larger pores.

**Figure 4:**
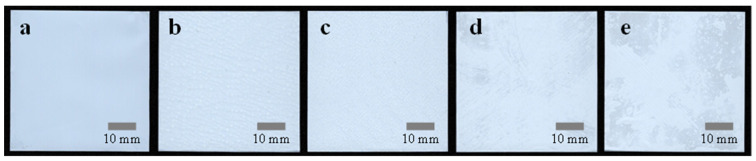
Digitally scanned images of separators: (**a**) bare and PVDF-HFP-coated separators with concentrations of (**b**) 10.0, (**c**) 12.5, (**d**) 15.0, and (**e**) 17.5 wt% followed by coagulation in DI water.

In case of the PVDF-HFP coating, the concentrations of 10.0, 12.5, 15.0, and 17.5 wt% were used for the R2R gravure coating. As the carrier film was maintained at a constant speed, a thicker PVDF-HFP film was achieved than the homopolymer PVDF film [32,33]. The digitally scanned images of the resulting PVDF-HFP-coated separators are shown in Figure 4. In contrast to the bare PE separator shown in Figure 4a, the PVDF-HFP-coated separators in Figure 4b–e exhibit an uneven surface. This unevenness can be attributed to the variation in the coating process, including the material distribution and the wetting/drying characteristics of the R2R coating process. Nevertheless, it is evident that this unevenness is decreased compared to the homopolymer PVDF coating. It appears that the thicker PVDF-HFP film serves as a mechanical support for the freestanding PE separators.

**Figure 5:**
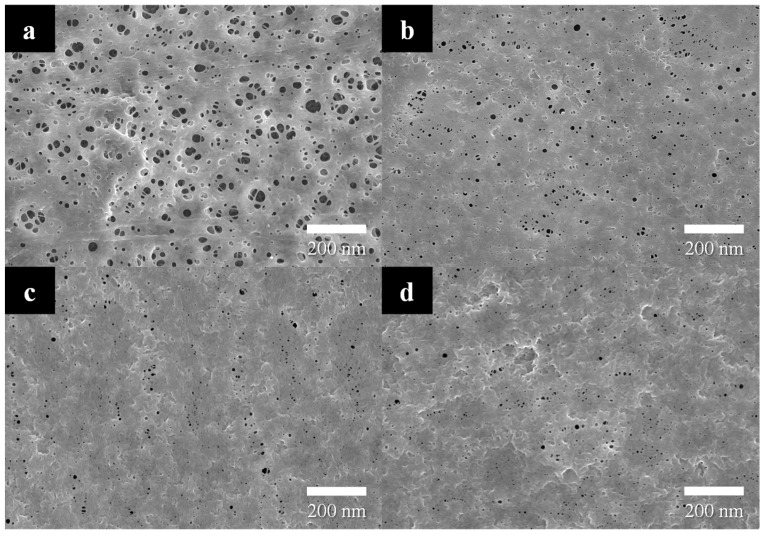
FE-SEM images of the PVDF-HFP-coated separators with concentrations of (**a**) 10.0, (**b**) 12.5, (**c**) 15.0, and (**d**) 17.5 wt% followed by coagulation in the DI water.

FE-SEM images of the PVDF-HFP-coated separators are displayed in Figure 5. In comparison to the fibrous structure observed in the bare separator as shown in Figure 3a, all the PVDF-HFP-coated separators exhibited round pores albeit with different sizes. In Figure 5a, PE polymer fibers are shown though the pores formed by means of NIPS. In contrast, the other PVDF-HFP-coated separators in Figure 5b–d do not show any PE fibers, primarily due to the thickness of the PVDF-HFP and the smaller pore size. Since the solute solidifies completely, the NIPS process takes place rapidly across the surface. However, in thicker PVDF-HFP film, the NIPS process is retarded, since it takes a longer time for the liquids to exchange during NIPS due to the obstruction by the solidified solutes. This results in fewer and smaller pores during solvent exchange. The higher the PVDF-HFP content, consequently, the smaller and fewer pores generated [34]. When the porosity becomes too low, and/or the mean pore size decreases, ion transfer through the separator is limited, resulting in reduced specific battery power. This can potentially compromise the mechanical strength of the separator and increase the risk of inner electrical short circuits [35]. A faster solvent exchange rate results in more and larger pores, which can be achieved either by decreasing the solute concentration or using the nonsolvent that is more miscible with NMP.

### 3.3. Thermal Resistance

**Figure 6:**
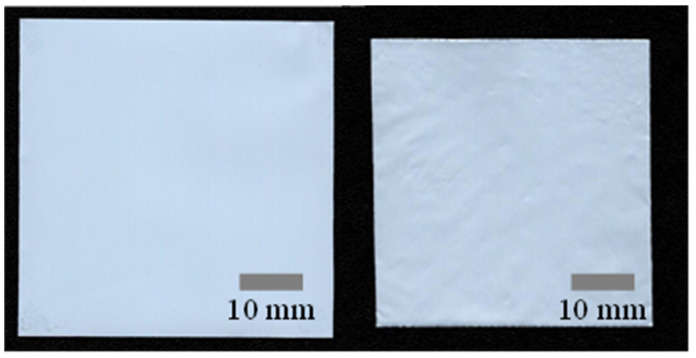
Digitally scanned images of the bare separator before (**left**) and after heat treatment (**right**).

The final goal of this study was to enhance the thermal stability of the separator through a straightforward polymer coating while maintaining the pores that serve as ion-transfer passages. Various studies have been conducted to assess the thermal resistance of coated separators. Of those, thermal resistance tests for simple polymer-coated separators were typically carried out at 130 °C for 30–60 min. However, tests for ceramic-coated separators were performed at higher temperatures, such as 150 °C [13,36,37]. While there were polymer-coated separators subjected to testing at 150 °C, they were typically the reinforced separators that had undergone processes such as photo-crosslinking with electron beam irradiation [9]. For this study, we chose to conduct the thermal resistance test at 130 °C for 30 min. Figure 6 displays the digitally scanned images of the bare PE separator before (left) and after the thermal resistance test (right). It is evident that the area is significantly decreased due to the natural thermal contraction of the polymer [38,39]. The digitally scanned images revealed 343,808 and 274,557 pixels for the area corresponding to the separator before and after thermal resistance test, respectively. As a result, the shrinkage rate was calculated as 20.1%.

**Figure 7:**
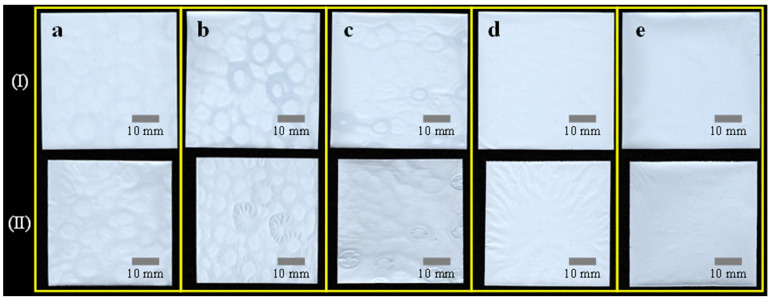
Digitally scanned images of the PVDF-(10 wt%)-coated separators before (row **I**) and after heat treatment (row **II**) after NIPS in a methanol concentration of (**a**) 0, (**b**) 25, (**c**) 50, (**d**) 75, and (**e**) 100%.

**Figure 8:**
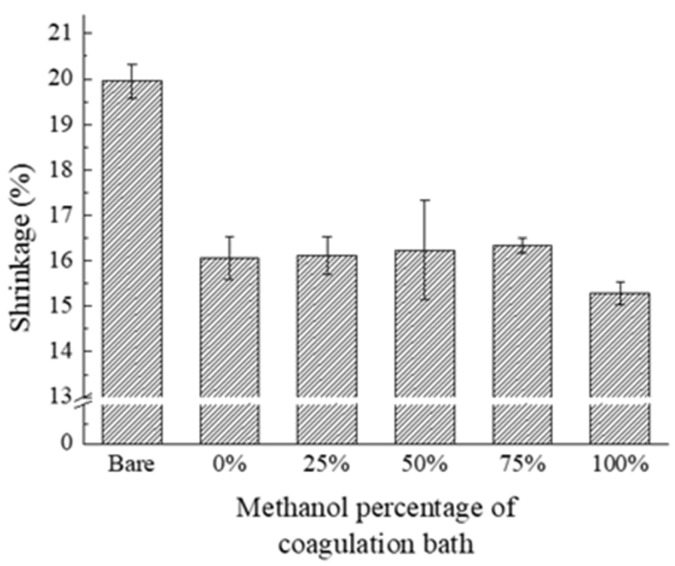
Shrinkage rate of the PVDF-(10 wt%)-coated separators after a heat resistance test.

The digitally scanned images of the homopolymer-PVDF-coated separators before (row I) and after the thermal resistance test (row II) are displayed in Figure 7. It is evident that the coated separators in row II have significantly shrunk. It is noted that the uneven surface has deteriorated, resulting in wavy shapes after thermal treatment. It would be a potential source of thermal stress and stress concentration in the battery separator, which may have adverse effects during battery operation. To quantitively compare shrinkage rate, the number of image pixels corresponding to the area of separators was counted and is shown in Table 3. In addition, the shrinkage rate was displayed in Figure 8 in terms of the methanol concentration in the coagulation solution. The homopolymer-PVDF-coated separators exhibit shrinkage rates of 16.1, 16.1, 16.2, 16.3, and 15.3%, which are considerably lower than the bare separator, presumably due to the coated PVDF film. However, the shrinkage rate does not vary significantly in response to changes in methanol content, except for the case of 100% methanol in NIPS. As shown in Figure 3, the methanol content significantly affects the number and size of pores. With differences in the pore size and number at methanol concentration of 0, 25, 50, and 75%, nevertheless, the thermal shrinkage remained relatively constant at around 16%. The reason for the reduction in the shrinkage rate from 20.1% in the pure PE separator to approximately 16% is the effect of the PVDF loading in the coating process. As long as the pores are well distributed (0~75%), they do not significantly influence thermal shrinkage. However, it is noted that the shrinkage rate is significantly reduced to 15.3% at 100% methanol concentration, presumably due to the fibrous structure of PVDF itself. Along with the fibers in the PE separator, fibrous PVDF contributes to enhancing mechanical stiffness, resulting in a lower thermal shrinkage rate.

**Figure 9:**
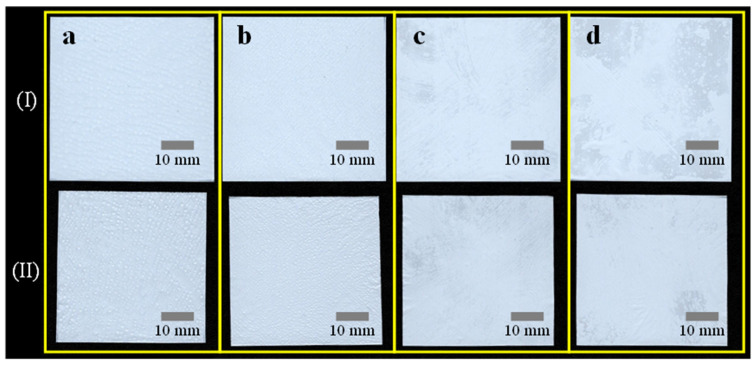
Digitally scanned images of the PVDF-HFP-coated separators before (row **I**) and after heat treatment (row **II**): coating solution concentration of (**a**) 10.0, (**b**) 12.5, (**c**) 15.0, and (**d**) 17.5 wt% followed by coagulation in DI water.

**Figure 10:**
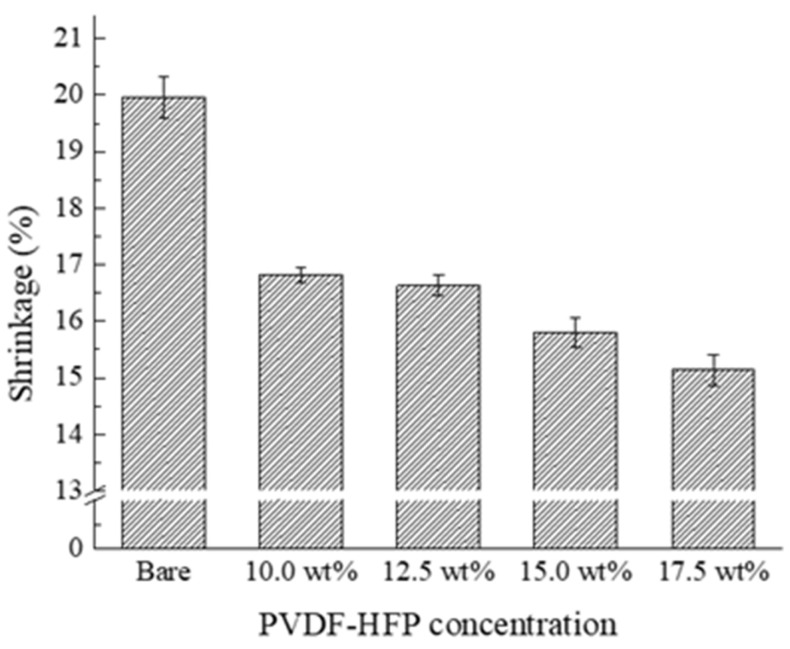
Shrinkage rate of the PVDF-HFP-coated separators after heat treatment.

The digitally scanned images of copolymer-PVDF-HFP-coated separators before (row I) and after the thermal resistance test (row II) is displayed in Figure 9. Similar to the homopolymer coating, the copolymer-PVDF-HFP-coated separators experienced some shrinkage after the thermal resistance test. However, fewer wrinkles and wavy shapes are observed compared to the homopolymer coating. The number of image pixels corresponding to the area of PVDF-HFP separators was also counted and is presented in Table 4. In addition, the shrinkage rate was displayed in terms of the concentration of PVDF-HFP in the coating solution in Figure 10. The copolymer-PVDF-HFP-coated separators exhibited shrinkage rates of 16.8, 16.6, 15.8, and 15.1%, which were lower than the bare separator, presumably due to the presence of the coated PVDF-HFP film. It is obvious that a lower shrinkage rate is achieved at a higher concentration of PVDF-HFP. It is interesting to note that a higher shrinkage rate is observed at 10 and 12.5 wt% of PVDF-HFP than at 10 wt% of PVDF coagulated in DI water/methanol. This can be attributed to the mechanical flexibility of the amorphous nature of the HFP, in contrast to the crystal nature of the VDF [40]. At concentrations of 15 and 17.5 wt%, however, a lower thermal shrinkage rate than homopolymer PVDF is found since more PVDF-HFP is coated on the separator. Even in the ultimate case of the fibrous structure of the PVDF, it exhibits higher thermal shrinkage than the PVDF-HFP coating with 17.5 wt% concentration.

## 4. Conclusions

The PE battery separator was coated with PVDF polymers using an R2R coating scheme to enhance the thermal stability. The adequately cut PE separators were affixed onto the PET carrier film, which was controlled to have a tension force of 9 kgf and a speed of 1 m/min. After gravure coating, the coated separator was continuously transported into a coagulation bath for NIPS, followed by drying. The morphology of the coated separators was characterized, revealing well-formed pores. The methanol contents in the coagulation bath influenced the structure of the pores formed in NIPS. The thermal resistance test demonstrated a significant reduction in the thermal shrinkage rate when the homopolymer PVDF was coated. However, the size and number of pores had little effect on changes in the thermal resistance when the pores were well distributed. Furthermore, copolymer PVDF-HFP was used to increase the thickness of the PVDF layer, followed by NIIPS with water as the nonsolvent. As the thickness of the PVDF-HFP increased, the thermal resistance decreased to 15.1%. The characteristics of the pores, including their number and size, should be carefully balanced with the enhancement of the thermal stability for battery performance. The choice of coating material and design of R2R NIPS play crucial roles in improving the functionality and thermal stability of the battery separator. The R2R coating proves to be an effective method for coating battery separators and can be seamlessly incorporated into the manufacturing process of the separators.

## Figures and Tables

**Table 1:** Hansen solubility parameters of the materials used in this study [3,4].

Material	$\delta_{d}$	$\delta_{p}$	$\delta_{h}$
PVDF	17.20	12.50	9.20
N-methyl-2-pyrrolidone (NMP)	18.00	12.30	7.20
Water	6.00	15.30	16.70
Methanol	7.42	6.00	10.90

**Table 2:** R values between the paired materials.

Pair of Materials	R
PVDF~NMP	2.57
PVDF~Water	23.79
PVDF~Methanol	20.68
NMP~Water	25.99
NMP~Methanol	22.39

**Table 3:** The number of pixels in the digitally scanned images of PVDF-coated separators before and after the thermal resistance test.

The Contents of Methanol in Coagulation Solution (%)	The Number of Pixels in Images		Average of Shrinkage Rate (%)
	Before Thermal Test	After Thermal Test	
0	344,235	288,778	16.1
	344,362	287,482	
	348,982	294,623	
25	349,691	294,288	16.1
	337,833	284,104	
	352,164	293,669	
50	348,593	294,888	16.2
	348,330	287,498	
	343,957	289,430	
75	347,599	290,477	16.3
	350,537	292,864	
	339,601	284,761	
100	349,647	297,002	15.3
	340,814	287,786	
	349,784	296,393	

**Table 4:** The number of pixels in the digitally scanned images of the PVDF-HFP-coated separators before and after the thermal resistance test.

The Concentration of PVDF-HFP in Coating Solution (wt%)	The Number of Pixels in Images		Average of Shrinkage Rate (%)
	Before Thermal Test	After Thermal Test	
10.0	349,207	290,371	16.8
	351,605	292,917	
	345,130	286,673	
12.5	348,094	290,186	16.6
	349,270	290,566	
	350,583	292,922	
15.0	348,842	294,054	15.8
	344,439	288,990	
	345,627	291,739	
17.5	346,180	294,262	15.1
	347,949	295,925	
	346,532	292,946	

## Data Availability

Data presented in this study are available on request from the corresponding author.
